# Percutaneous Cryoablation for the Treatment of Medically Inoperable Stage I Non-Small Cell Lung Cancer

**DOI:** 10.1371/journal.pone.0033223

**Published:** 2012-03-08

**Authors:** Yoshikane Yamauchi, Yotaro Izumi, Kohei Hashimoto, Hideki Yashiro, Masanori Inoue, Seishi Nakatsuka, Taichiro Goto, Masaki Anraku, Takashi Ohtsuka, Mitsutomo Kohno, Masafumi Kawamura, Hiroaki Nomori

**Affiliations:** 1 Department of Surgery, School of Medicine, Keio University, Tokyo, Japan; 2 Department of Diagnostic Radiology, School of Medicine, Keio University, Tokyo, Japan; 3 Department of Surgery, Teikyo University School of Medicine, Tokyo, Japan; National Taiwan University Hospital, Taiwan

## Abstract

**Background:**

To evaluate the midterm results of percutaneous cryoablation for medically inoperable stage I non-small cell lung cancer.

**Methodology/Principal Findings:**

Between January 2004 and June 2010, 160 patients underwent computer tomography guided percutaneous cryoablation for lung tumors at our institution. Of these patients, histologically proven stage I lung cancer patients with more than one year of follow-up, were retrospectively reviewed. All of these patients were considered to be medically inoperable with Charlson comorbidity index of 3 or greater. Follow-up was based primarily on computed tomography. There were 22 patients with 34 tumors who underwent 25 sessions of cryoablation treatment. Complications were pneumothoraces in 7 treatments (28%, chest tube required in one treatment), and pleural effusions in 8 treatments (31%). The observation period ranged from 12–68 months, average 29±19 months, median 23 months. Local tumor progression was observed in one tumor (3%). Mean local tumor progression-free interval was 69±2 months. One patient died of lung cancer progression at 68 months. Two patients died of acute exacerbations of idiopathic pulmonary fibrosis which were not considered to be directly associated with cryoablation, at 12 and 18 months, respectively. The overall 2- and 3-year survivals were 88% and 88%, respectively. Mean overall survival was 62±4 months. Median overall survival was 68 months. The disease-free 2- and 3-year survivals were 78% and 67%, respectively. Mean disease-free survival was 46±6 months. Pulmonary function tests were done in 16 patients (18 treatments) before and after cryoablation. Percentage of predicted vital capacity, and percentage of predicted forced expiratory volume in 1 second, did not differ significantly before and after cryoablation (93±23 versus 90±21, and 70±11 versus 70±12, respectively).

**Conclusions/Significance:**

Although further accumulation of data is necessary regarding efficacy, cryoablation may be a feasible option in medically inoperable stage I lung cancer patients.

## Introduction

Surgical resection is currently the standard treatment for stage I non-small cell lung cancer (NSCLC). However, in patients who are medically inoperable due to significant comorbidities, other treatment modalities need to be considered. The non-surgical management of early stage lung cancer is currently an expanding field. These include stereotactic body radiation therapy (SBRT) and thermal ablative procedures such as radiofrequency ablation (RFA) and microwave ablation [1], [2], [3], [4], [5], [6]. Percutaneous cryoablation is also currently evolving as a minimally invasive, and potentially effective, local treatment for lung tumors [7], [8], [9], [10]. This procedure, mostly used when surgical resection is contraindicated, is currently under evaluation as a potential complementary therapy for patients with primary lung cancers as well as metastatic lung tumors. We have, to date, treated more than 300 lung tumors in more than 200 patients with acceptable feasibility and efficacy. Of these patients, in the present study, we retrospectively analyzed the midterm outcomes of stage I NSCLC patients treated with cryoablation.

## Materials and Methods

### Ethics

This study protocol was approved by Keio University institutional review board (approval ID: 14–23). Written informed consent was obtained from each participant in accordance with the Declaration of Helsinki.

### Selection of patients

Between January 2004 and August 2010, 160 patients underwent cryoablation for lung tumors at our institution. Of these patients, we retrospectively reviewed our experience with cryoablation for the primary treatment of stage I NSCLC in medically inoperable patients, with more than one year of follow-up. The tumors which presented as multiple tumors in one patient, were clinically considered as synchronous or metachronous primary lung cancers to be eligible for this study. Some of these patients have been reported previously [7], [9].

Prior to considering cryoablation, patients with histologically diagnosed NSCLC were routinely staged with chest-to-pelvis computed tomography (CT), brain magnetic resonance imaging (MRI) or CT, and most of the patients also underwent a positron emission tomographic (PET) scan. Bone scintigraphy was done if PET scan was not performed. Patients with hilar or mediastinal lymph nodes greater than 1 cm in the shortest axis, a positive PET scan result, or both, underwent endobronchial ultrasonography guided needle biopsy, or mediastinoscopy.

The inclusion criteria for this study were patients who were considered medically inoperable because of risks such as impaired cardiac function, poor pulmonary function, and/or other comorbidities, i.e., Charlson comorbidity index (CCI) [11] > = 3. The patients' desires to avoid surgery in association with their medical comorbidities were also accounted for. The exclusion criteria were as follows: (1) Eastern Cooperative Oncology Group (ECOG) score of 2 or more. (2) Platelet count of less than 50,000/µL. (3) Prothrombin time international normalized ratio of more than 1.5. (4) No suitable way for the insertion of probes due to interference by major vasculatures, airways or mediastinal structures. (5) Incapable of cooperation during the cryoablation procedure. All patients were evaluated by representatives from pulmonologists, interventional radiologists, and thoracic surgeons to determine inoperability and suitability for cryoablation.

### Cryoablation procedure

The procedure of percutaneous cryoablation was performed under local anesthesia as previously described [7]. Under a multidetector-row CT scanner with multi-slice CT fluoroscopy functions (Aquilion 64; Toshiba Med. Co. Ltd., Tokyo, Japan), using an outer insertion sheath, a 1.7-mm-diameter cryoprobe (CRYOcare Cryosurgical Unit; Endocare, Irvine, CA) was inserted into the targeted nodule under fluoroscopic CT guidance. Multiple probes were simultaneously inserted if the ablation margin was considered to be insufficient with just one probe. The cryoprobe uses high-pressure argon and helium gases for freezing and thawing, respectively, based on the Joule-Thompson principle. Cryoablation consisted of three cycles of freezing, 5, 10, and 10 minutes each. The tip of the cryoprobe reaches approximately −130°C during freezing. This was followed by thawing until the temperature of the cryoprobe reached 20°C, and then a third cycle of freezing (10 minutes) followed by thawing. Fibrin glue was infused into the outer sheath at the time of cryoprobe removal to reduce the risks of hemothoraces and pneumothoraces. Whole lung CT scans were taken at the ends of each of the procedures. Chest radiographs were also taken two hours after, the next day, and the day after each of the procedures to check for complications such as hemothoraces or pneumothoraces. The patients were discharged on the second postoperative day if there were no complications.

### Follow-up after cryoablation

Follow-up chest-to-pelvis CT scans with contrast enhancement were carried out at 1-month and then at 3 to 6 months intervals after cryoablation. We confirmed local progression when there was a continuous focal or diffuse enlargement of the ablated lesion on CT. Furthermore, even when no enlargement was seen, we regarded it as local progression if the size of partial enhancement in the tumor continuously increased. As for the detection of distant metastases, brain MRI or CT was done every 3 to 6 months. PET scan or bone scintigraphy was done if considered to be necessary.

### Pulmonary function test

Pulmonary function test was done in patients who could adequately perform the test, before, and 3 to 6 months after cryoablation.

### Statistical methods

Local tumor progression–free intervals, and overall and disease-free survivals, were calculated with the Kaplan–Meier method. Pulmonary function tests were compared with the paired t test. The statistical software package SPSS 17.0 (SPSS Inc, Chicago, Ill) was used for all analyses. P values smaller than 0.05 was considered to be statistically significant.

## Results

During the study period, 22 patients with 34 tumors underwent 25 sessions of lung cryoablation treatments for clinical stage I NSCLC. These patients were retrospectively reviewed. None of the patients had mediastinal or hilar lymph nodes greater than 1 cm in the shortest axis, or a positive PET scan result of the mediastinal or hilar lymph nodes. Fifteen patients had single tumors, which were all treated in one session. Three patients had 2 tumors. The 2 tumors were found synchronously in all 3 patients, and were treated as one session per patient. Four patients had 3 tumors. In 2 of these patients, the 3 tumors were found synchronously, and were treated as one session per patient. In both of the remaining 2 patients, 2 tumors were synchronous and one was metachronous. The 2 synchronous tumors were treated in one session in each of the patients. The metachronous tumors were treated as another session in both patients. One patient had 4 tumors. Two of these tumors were found synchronously and were treated in one session. Other 2 metachronous tumors were found at the same time, and were treated in one session. The patient and tumor characteristics are described in Table 1. The mean maximal tumor diameter was 1.4±0.6 cm (range 0.5–3.0 cm). More than half of the patients had a past history of resection for another lung cancer. Majority of tumors were adenocarcinomas. Nine patients had more than one tumor, which were considered to be synchronous, or metachronous primary lung cancers. The number of probes used was 1 in 20 tumors, 2 in 13 tumors, and 3 in 1 tumor. Eight patients (36%) had significant cardiac or vascular disease that put them at high risk for surgical resection. Limited pulmonary function was the predominant determinant of medical inoperability in 6 patients (27%). Four of these patients were on oxygen therapy. Other comorbidities included renal dysfunction, liver dysfunction, and concomitant malignancies. Average CCI was 5±3, range 3 to 15.

**Table 1:** Patient and tumor characteristics.

Sex (female/male)	11∶11
Age (y, median)	72
Lesion size (cm)	
Mean ± SD	1.4±0.6
Range	0.5–3.0
Previous lung cancer resection	12 (57%)
Patients with more than one lesion	9 (43%)
ECOG PS score	
0	10
1	12
Charlson comorbidity score	
Mean ± SD	5.0±2.9
Range	3–15
Clinical stage	
T1aN0	29
T1bN0	5
Histologic type	
Squamous	2
Adenocarcinoma	30
Large cell carcinoma	1
Non-small cell lung carcinoma	1
Subsolid nodules	12

The most common complications of cryoablation were pneumothoraces, minor hemoptyses, and pleural effusions. Pneumothoraces were seen in 7 treatments (28%). Pleural effusions were seen in 8 treatments (31%). Minor hemoptyses were seen in 6 patients (24%). Chest tube insertion was required in one patient with pneumothorax. All other complications resolved with observation only.

**Figure 1:**
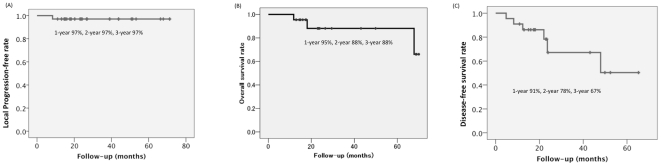
Kaplan–Meier estimate curves of (A) local progression-free interval after cryoablation, (B) overall survival after cryoablation, and (C) disease-free survival after cryoablation.

The observation period ranged from 12–68 months, average 29±19 months, median 23 months. Local tumor progression after cryoablation was observed in one tumor (3%) which was a squamous cell carcinoma 1.6 cm in size. Local failure was recognized as progressive enlargement of the ablated region at 8 months after cryoablation. At this time, no other metastases were observed. The local recurrent tumor was re-cryoablated. Four months after re-cryoablation, the patient developed an upper respiratory infection, which lead to an acute exacerbation of the underlying idiopathic pulmonary fibrosis (IPF). The patient subsequently died of the acute exacerbation. At this point, local control was maintained. Overall, the mean local tumor progression-free interval was 69±2 months. Median local tumor progression-free interval was not reached (Figure 1A).

So far 3 patients (14%) have died. One patient was the patient described above. Another patient died of lung cancer 68 months after cryoablation. This patient developed multiple systemic metastases whereas local control was maintained. This patient received chemotherapy one year after cryoablation because distant metastases were detected. The remaining one patient died of acute exacerbation of IPF 18 months after cryoablation. In this case, the acute exacerbation of IPF occurred immediately after chemotherapy for concomitant liver cancer, and was not considered to be directly associated with lung cryoablation. There are 2 patients who have received chemotherapy and are alive. One patient developed multiple lung metastasis 48 months after cryoablation and have received systemic therapy with gefitinib. Local control was maintained in this patient. The other patient developed multiple systemic metastases 4 months after cryoablation. Local control was maintained. This patient received systemic chemotherapy after detection of distant metastases. The overall 2- and 3-year survivals were 88% and 88%, respectively. Mean overall survival was 62±4 months. Median overall survival was 68 months (Figure 1B). Five patients are alive with lung cancer. The disease-free 2- and 3-year survivals were 78% and 67%, respectively. Mean disease-free survival was 46±6 months. Median disease-free survival was not reached (Figure 1C).

The patterns of recurrences other than local recurrence were as follows: Recurrence only in the ipsilateral thorax was seen in 1 patient, which was lung metastases. Needle-tract disseminations or pleural recurrences have not been detected so far in any of the patients. Distant metastases were seen in 5 patients. These included metastases to contralateral thoraces, lumbar vertebra, ribs, and brain. Treatments for these patients included chemotherapy, radiation, and gamma-knife.

**Figure 2:**
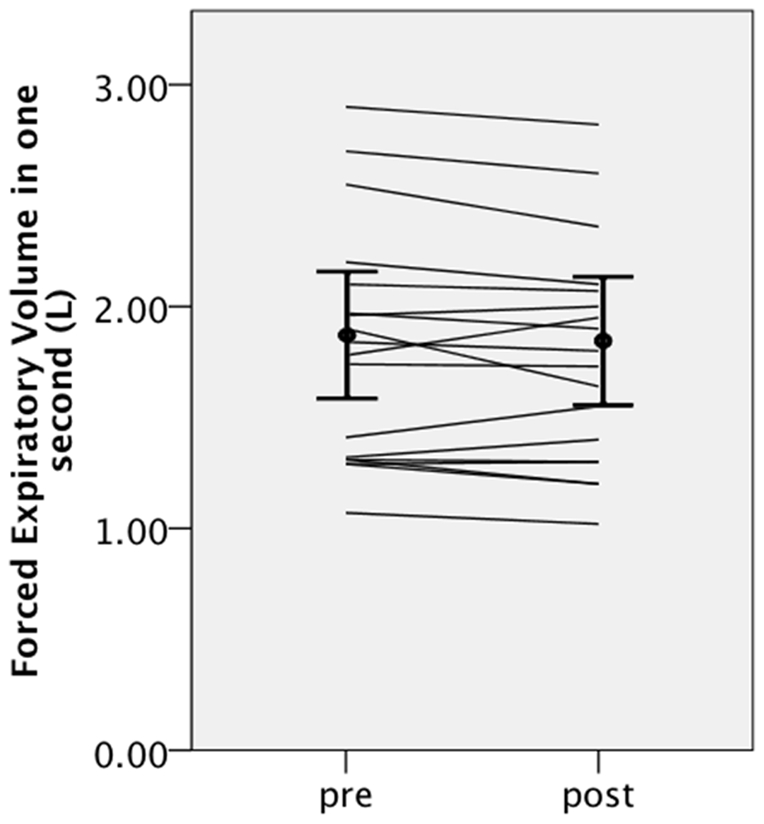
Individual changes in forced expiratory volume in 1 second, and the average ± standard deviations before and after cryoablation.

Pulmonary function was evaluated in 16 patients (18 treatments) before and 3 to 6 months after cryoablation. There were no significant differences before and after cryoablation in vital capacity (2.72±0.82 L versus 2.64±0.74 L, p = 0.19), percentage of predicted vital capacity (93±23% versus 90±21, p = 0.11), forced expiratory volume in 1 second (1.81±0.53 L versus 1.77±0.50 L, p = 0.14) (Figure 2), and percentage of forced expiratory volume in 1 second (70±11% versus 70±12%, p = 0.95).

## Discussion

There is accumulating evidence that RFA is a safe and feasible treatment option for the treatment of inoperable stage I NSCLC. There is one report in which the results of cryoablation for stage I lung cancer is included among the results of RFA and sublobar resections [10]. But to our knowledge, this is the first report which specifically focuses on cryoablation in patients with medically inoperable stage I NSCLC. In the present study, cryoablation was done safely in all patients. Reduction in pulmonary function after cryoablation was also minimal in this study, although the pulmonary function test was done mostly in patients with relatively good pulmonary functions who could adequately perform the test. The incidences of the most common complications, which were pneumothoraces, and pleural effusions, were comparable to those previously reported for RFA [5], [6], [12], [13], [14].

The reported local control rates for RFA treatment of inoperable stage I NSCLC ranged from 58 to 69% [5], [6], [12], [13], [14]. The local control rate was slightly higher in the present study (97%), presumably because in our study the tumors were 3 cm or less, actually mostly 2 cm or less, whereas previous RFA studies included tumors which were 4 cm or less. As for the one patient with local recurrence, we speculate that the primary cause of local progression was insufficient margin of ablation. Although 2 probes were used in this case, it was difficult to delineate the relationship between the margin of ablation and the margin of the tumor on CT because of the underlying IPF. We consider that further accumulation of experience is necessary to improve treatment outcomes in such cases. The overall and disease-free survival at 3 years were better than that previously reported for RFA [5], 88% and 67% versus 47% and 39%, respectively. This was also presumably because in our study the tumors were 3 cm or less, whereas the previous RFA study included tumors which were 3–4 cm. In our study, there were 6 patients with disease progression other than local recurrence, but the number of patients was too small to evaluate if there is any characteristic pattern of disease progression after lung cancer cryoablation.

Determination of medical inoperability is critically important and should be assessed by an interdisciplinary team. A patient should not be judged as inoperable by one factor alone, such as poor pulmonary function. Therefore the assessment of medical operability requires a comprehensive evaluation of multiple factors in the patient. To this end, the group of patients in the present study all had significant associated comorbidities, with CCIs of > = 3. This score has been validated in surgically resected patients with lung cancer [15], [16]. In these reports, multivariate analysis showed that a CCI > = 3 was a significant predictive factor of increased risk of major complications. In the current study, the patients who underwent cryoablation were elderly (median age, 72 years), had significant comorbidities (mean CCI, 5), and therefore, were considered to represent a high-risk population for surgery. Although further follow-up is needed, so far only one patient in this study has died of lung cancer, and other 2 patients have died of their comorbidities. This result suggests that minimally invasive treatment options such as cryoablation may actually be appropriate for patients with substantial comorbidities.

In terms of efficacy, there is evidence to suggest that cryoablation may result in improved local control in comparison to RFA in renal tumors [17], but to our knowledge there are no studies comparing the two modalities in lung tumors. Since this is a retrospective, observational study with a relatively short follow-up in a limited number of highly selected patients subjected to multiple biases, further studies are necessary to more appropriately address the outcomes of cryoablation in comparison to RFA for early stage lung cancer. SBRT is also evolving to be a promising treatment option for early stage lung cancer, with remarkable improvements in efficacy and safety. The indications for SBRT and ablative procedures are expected to be very similar, and further studies are necessary to delineate the strengths and weaknesses of each of these modalities, which may be complementary rather than mutually exclusive.
